# Patient Phenotyping for Atopic Dermatitis with Transformers and Machine Learning

**DOI:** 10.1101/2023.08.25.23294636

**Published:** 2023-08-28

**Authors:** Andrew Wang, Rachel Fulton, Sy Hwang, David J. Margolis, Danielle L. Mowery

**Affiliations:** 1 Department of Computer and Information Science, School of Engineering and Applied Sciences, University of Pennsylvania, Philadelphia, PA; 2 Lankenau Medical Center, Dermatology Services, Wynnewood, PA; 3 Institute for Biomedical Informatics, Perelman School of Medicine, University of Pennsylvania, Philadelphia, PA; 4 Department of Dermatology, Perelman School of Medicine, University of Pennsylvania, Philadelphia, PA; 5 Department of Biostatistics, Epidemiology & Informatics, Perelman School of Medicine, University of Pennsylvania, Philadelphia, PA

**Keywords:** patient phenotyping, atopic dermatitis, machine learning, natural language processing

## Abstract

**Background:**

Atopic dermatitis (AD) is a chronic skin condition that millions of people around the world live with each day. Performing research studies into identifying the causes and treatment for this disease has great potential to provide benefit for these individuals. However, AD clinical trial recruitment is a non-trivial task due to variance in diagnostic precision and phenotypic definitions leveraged by different clinicians as well as time spent finding, recruiting, and enrolling patients by clinicians to become study subjects. Thus, there is a need for automatic and effective patient phenotyping for cohort recruitment.

**Objective:**

Our study aims to present an approach for identifying patients whose electronic health records suggest that they may have AD.

**Methods:**

We created a vectorized representation of each patient and trained various supervised machine learning methods to classify when a patient has AD.

**Results:**

The most accurate AD classifier performed with a class-balanced accuracy of 0.8036, a precision of 0.8400, and a recall of 0.7500 when using XGBoost (Extreme Gradient Boosting).

**Conclusions:**

Creating an automated approach for identifying patient cohorts has the potential to accelerate, standardize, and automate the process of patient recruitment for AD studies, therefore reducing clinician burden and informing knowledge discovery of better treatment options for AD.

## Introduction

### Background

Atopic dermatitis (AD) is a common skin disease with a population prevalence of between 10% and 20% [1–4]. It is often diagnosed in early childhood, but onset can occur at any age [1–4]. Symptoms of AD include inflamed, red, irritated, and itchy skin and can cause significant physical and emotional distress. AD is often associated with other allergic illnesses including asthma, seasonal allergies, and food allergies [1–3,5,6].

AD is thought to be associated with skin barrier dysfunction and immune dysregulation [3]. AD has also been associated with genetic variation as well as environmental factors [3]. Classic treatment for AD has included the use of moisturizers, topical steroids, and other topical anti-inflammatory agents [7]. However, in the past few years, there have been significant treatment advances, which include systemic agents that alter immune function such as dupilumab. Therefore, due to the widespread nature of AD, the need for improved knowledge of the natural history of AD, the need to understand the efficacy of new treatments, and the need to develop new treatments, there is an urgent need to understand the clinical course of individuals with AD. However, identifying appropriate cohorts of patients for medical studies can be difficult and time consuming. Because AD is so common as well as diagnosed and managed by many different clinicians in varying healthcare settings, a potential source population would be patients from a health system’s electronic health records (EHRs) [8]. Investigators often ascertain a patient’s illness using International Classification of Disease (ICD) hospital billing codes as recorded during routine office visits. However, it has been previously demonstrated that reliance on ICD codes is not an accurate method for the ascertainment of AD study cohorts [8,9]. Furthermore, epidemiologic studies have used different methods and algorithms including the UK Working Party (UKWP) diagnostic criteria and the Hanifin and Rajka (HR) criteria [10,11]. Investigators attempting to conduct clinical trials and observational studies have also relied on manual, large-scale chart review, a process that is inefficient, slow, and tedious [8]. This motivates the need for a standard method to accurately, automatically, and efficiently identify potential patient cohorts from their text medical records via natural language processing (NLP) and machine learning (ML) techniques.

### Prior Work

Previously, researchers have aimed to phenotype patients with AD using EHR data. In particular, Gustafson et al. trained a logistic regression model with lasso regularization to identify cases of AD from the Northwestern Medical Enterprise Data Warehouse (NMEDW) which contained both structured data (ICD-9/ICD-10 codes, medication prescriptions, and lab results), as well as unstructured data (clinician notes from patient encounters) [9]. A gold standard diagnosis was assigned to each patient in their dataset by two rheumatologists following a chart review when using the UK Working Party (UKWP) criteria, and (alternatively) when using the Hanifin and Rajka (HR) criteria.

Although similar, our work differs in the following ways: 1) we survey a wide range of supervised machine learning algorithms as opposed to only using lasso regularized logistic regression, 2) we provide meaning and context of sentences to our machine learning algorithms by embedding sentences with transformers as opposed to feeding counts of different phrases into our algorithms, and 3) we performed an ablation study of processing methods to compare the impact on performance in using a probability-based vs binary label of whether each patient meets various AD diagnostic criteria when creating a vector to represent each patient for input to our final AD patient phenotyping algorithms.

## Methods

### Overview

To predict whether a patient may qualify as a subject for an AD study based on their electronic health record, we first assigned patients in our dataset to either the training or testing set. Then, for each patient, we aggregated the text from their EHR and constructed a vector representation of clinical features indicative of AD according to the UKWP criteria. Lastly, we leveraged our vectorized patient representations to train several machine learning classifiers to predict whether each patient has AD. In the following sections, we detail this process.

### Dataset Creation

We initially sampled 2,000 patients and their clinical records from Epic Clarity, Penn Medicine’s EHR database. We selected Penn Medicine patients who were diagnosed with a subset of AD-related ICD codes [8]. Of the 2,000 sampled patients, we identified 1,926 patients who had clinical notes for processing. We then de-identified these patient records according to the Safe Harbor method using PHIlter [12]. Each patient in the dataset was also manually reviewed and labeled according to the UK Working Party (UKWP) diagnostic criteria for AD. According to the UKWP criteria, in order to qualify as having AD, a patient must have an itchy skin condition along with 3 or more of the following: a history of flexural involvement, a history of asthma or hay fever, a history of dry skin, an onset of rash under the age of 2 years, or a visible flexural dermatitis. Our dataset was validated by two clinicians (a board-certified dermatologist (DJM) and a medical fellow (RF)), resulting in 137 patients with AD and 1,789 patients without AD.

### Training and Testing Split

We first assigned patients in our dataset to either the training or testing set. Due to the heavy class imbalance in our dataset, we under-sampled from the over-represented class of non-AD patients as part of our preprocessing, resulting in 137 patients in each class (AD and non-AD) from which we created our training (80%) and testing (20%) sets. We chose not to create a separate hyperparameter tuning set and instead applied cross validation for hyperparameter tuning on the training set due to the data scarce setting of our experiments.

### Vector Representation for AD Classification

**Figure 2.**
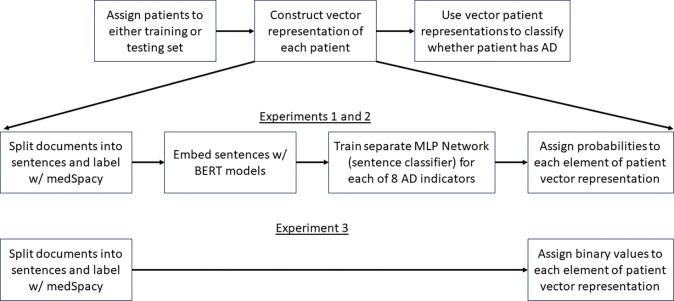
AD Phenotyping pipeline across all 3 experiments

Next, we created a vector representation for each patient. We performed 3 experiments to compare different methods of creating each patient’s vector representation (Figure 2).

#### Description of Patient Vector Representation

Each patient’s vector representation is 8 elements long, where each element of the vector is representative of whether the patient fulfills a different AD diagnosis criteria based on the UKWP criteria as well as clinician feedback (Table 1).

In experiments 1 and 2, elements 1 through 8 of each patient’s vector represent the highest probability that any sentence in the patient’s EMR mentions 1) AD or synonyms of AD, 2) keywords that suggest hay fever allergies, 3) keywords that suggest atopic allergies, 4) keywords that suggest eczema or rashes, 5) keywords that indicate dry or itchy skin, 6) keywords denoting non-asthma medications, 7) keywords suggesting the presence of asthma, and 8) keywords indicating the use of asthma medications. In experiment 3, instead of each element representing a probability, each element represents a binary value of whether there was at least 1 sentence in the corresponding patient record suggesting the presence of the corresponding AD indicator.

In the first two experiments, each patient’s vector elements represent probabilities (ranging from 0 to 1). Experiments 1 and 2 were performed to compare the use of two BERT models (BERT Base Uncased in experiment 1, and BioClinical BERT in experiment 2) for creating sentence embeddings used to train multi-layer perceptron (MLP) networks (or alternatively, sentence classifiers). A separate MLP network is trained for each element of the patient vector. Each MLP network is trained to distinguish sentences in one of the 8 AD indicator categories from sentences in all other categories. Furthermore, medSpacy was used to split documents into sentences and label each sentence with different categories. After each sentence classifier is trained, embeddings of all sentences in each patient’s full EHR are passed through each sentence classifier and an aggregation function (max operator) is used to assign a value to each element of each patient’s vector. Our goal in experiment 1, was to test the hypothesis that a BERT model pretrained on clinical text (BioClinical BERT) could outperform a BERT model trained on non-clinical text (BERT Base Uncased).

In experiment 3, each patient’s vector elements are binary (either 0 or 1). Each element corresponds to a diagnostic criteria and represents whether medSpacy was able to identify at least 1 sentence in the patient’s record with a keyword and affirming context that suggests the patient meets the corresponding diagnostic criteria. Our goal was to conduct an ablation study to test the hypothesis that an AD patient classifier leveraging BERT embeddings to create the patient vector representation will better discern whether a patient has AD than an AD patient classifier without BERT embeddings.

#### Preprocessing for Experiments 1–3

Before each experiment, we applied the same preprocessing steps to assign one or more labels to each sentence in our corpus of documents in both our training and testing sets. Each sentence can be labeled as applying to one, multiple, or none of the 8 AD indicators previously defined.

For each of the 8 diagnostic criteria, we first created a list of keywords and phrases (for each vector element) that suggested the presence of the corresponding diagnostic criteria. Next, we used medSpacy with the ConText algorithm to split each document into sentences and categorize each sentence [13]. Using medSpacy allows us to obtain sentences that suggest the presence of each of the 8 diagnostic criteria due to medSpacy’s use of regex and rules-based keyword matching. Furthermore, medSpacy’s implementation of the ConText algorithm allows us to discern between sentences that affirm from negated assertions. We define negated sentences for each AD indicator as sentences where the indicator is ruled out, sentences where the indicator is experienced by someone other than the patient, and sentences where the existence of the indicator is hypothetical [14–17].

After assigning one or more categorical labels to each sentence with medSpacy, we then performed 3 different experiments to create a vectorized representation of each patient.

#### Experiments 1 and 2 – Patient Vector Construction with BERT Embeddings

In experiments 1 and 2, we used pretrained BERT models to first generate embeddings of sentences in each category. We incorporated pretrained BERT models because these models have been trained on a much larger corpus than our existing dataset, and BERT provides a context sensitive embedding of text which other techniques such as bag of words don’t provide. Furthermore, we used BERT Base Uncased in experiment 1, and Alsentzer et. al’s BioClinical BERT in experiment 2 because we wanted to quantify how much of a difference in performance that using a model pretrained on clinical text can provide over a model that has not been pretrained on clinical text.

Using these embeddings, we trained a multi-layer perceptron (MLP) network to distinguish sentence embeddings in each category from sentence embeddings that aren’t in the corresponding category. Each of our MLP’s were trained with the following architecture: a fully connected input layer of shape 768 by 100, followed by a ReLU (Rectified Linear Unit) activation, further followed by a fully connected output layer of shape 100 by 2. We trained each of our MLP’s for 10 epochs with the cross-entropy loss function, the stochastic gradient descent (SGD) optimizer, a learning rate of 0.001, and a momentum value of 0.9. The final layer of each MLP can then be used to obtain the probability that any given sentence embedding comes from the category for which the MLP is being trained by passing the logits of the final layer to the softmax function.

We used the Rectified Linear Unit activation function as defined below, where x is the input to the ReLU function:

ReLU⁡(x)=max(0,x)

We also used the softmax function as defined below, where e is the standard exponential function, ${\vec{x}}_{i}$ is element at index i of the K element long input vector $\vec{x}$.


$$softmax(\vec{x}{)}_{i}=\frac{e^{x_{i}}}{\sum_{i=1}^{K}\;e^{x_{i}}}$$

We chose to embed our sentences once with pretrained BERT models, and then feed these saved embeddings to our MLP networks as opposed to adding a classification head (a linear layer) to the end of our pretrained BERT models. Although doing so only allows us to fine tune the weights in our MLP network (as opposed to also fine tuning the weights BERT uses to embed the sentences), doing so allows us to iterate over different experiments more quickly and with less computational power. In particular, we are able to 1) avoid the large computational expense of gradient calculations during backpropagation for all 12 layers of transformers used by BERT when fine tuning the model, 2) avoid the computational expense of repeatedly generating the same embeddings from BERT multiple times (if we chose to freeze the weights of BERT and only fine tune an added classification head/linear layer), and 3) iterate more efficiently over different hyperparameter combinations across different experiments with our MLP networks.

After training a separate MLP network for each of the 8 categories, we generated a vector representation for each patient where each of the 8 vector elements represents the highest probability that any given sentence in the patient record affirms the presence of the corresponding AD indicator. We accomplished this by iterating through all sentences in each patient’s full EHR and passing the sentence embedding through each of our 8 trained MLP networks to obtain 8 probabilities for each sentence corresponding to the probability that the sentence affirms each of the 8 AD indicators we previously selected. Then, for each patient and for each AD indicator, we kept the highest probability that any given sentence in the patient’s record affirms the presence of the AD indicator.

#### Experiment 3 – Patient Vector Construction without BERT Embeddings

In experiment 3, we generated each patient’s vector representation by assigning a 1 to each element of the patient vector if medSpacy with the ConText algorithm identified at least 1 sentence in the patient’s record that affirms or suggests the presence of the AD indicator for which the vector element corresponds to. Experiment 3 was conducted as an ablation study to quantify the performance benefit (if at all) of using contextual BERT text embeddings to generate probability scores that the patient meets various AD indicators.

### AD Phenotyping with Vector Representations

In all three experiments, after generating a vector representation for each patient, we collated each patient vector representation with the corresponding label our clinicians assigned the patient when validating the dataset. Then, we fed the vector patient representation and corresponding patient label through a variety of classification algorithms. These include logistic regression, support vector machines (SVM), decision trees, random forests, K nearest neighbors (KNN), Extreme Gradient Boosting (XGBoost), and Adaptive Boosting (AdaBoost). During training for each of the previously mentioned classifiers, we used 5-fold cross validation to determine the best set of hyperparameters to use (as opposed to creating a separate validation set) due to the data scarce setting of our experiments. We then used the selected hyperparameters to train each algorithm on the entire training set and evaluated performance on the testing set. In addition to using the previously mentioned classifiers, we also used the stacking algorithm provided by scikit-learn to obtain an ensemble prediction from the different classifiers [18]. To quantify performance, we calculated the accuracy, precision, and recall of each algorithm on the class balanced testing set.

We define accuracy, precision, and recall as follows, where TP is the number of true positives, TN is the number of true negatives, FP is the number of false positives, and FN is the number of false negatives:

$$Accuracy\;=\frac{TP\;+\;TN}{TP\;+\;TN\;+\;FP\;+\;FN}$$

$$Precision\;=\frac{TP}{TP\;+\;FP}$$

$$Recall\;=\frac{TP}{TP\;+\;FN}$$

## Results

### Performance of MLP Networks

As part of our AD Phenotyping pipeline, we trained various MLP networks to classify when a given sentence embedding indicates the presence of an AD indicator, and we compared performance of BioClinical BERT embeddings to BERT Base Uncased embeddings when training these MLP networks. In both cases, the classifier with the highest accuracy was the classifier for category 1 and the classifier with the lowest accuracy was the classifier for category 3.

Before training each of our MLP Networks, we first created a class-balanced training and testing set for each of our classifiers as shown in table 2. The same training and testing set was used for both experiment 1 (BioClinical BERT) and experiment 2 (BERT Base Uncased).

In experiment 1, the accuracies across AD indicator classifiers ranged from 0.7143 (classifier 3) to 0.9814 (classifier 1) as shown in table 3 below.

In experiment 2, the accuracies across AD indicator classifiers ranged from 0.7321 (classifier 3) to 0.9698 (classifier 1) as shown in table 4 below.

### AD Phenotyping with Patient Vector Representations

In experiment 1, we leveraged BioClinical BERT sentence embeddings to train various MLP networks to discern sentence embeddings in different AD indicator categories. Then, we applied these trained MLP networks (sentence classifiers) along with an aggregation function (max operator) to assign values to each element of each patient’s vector representation. Lastly, we used each patient’s vector representation with their validated label to train various ML algorithms. As shown in table 5, the accuracy of these ML algorithms ranges from 0.6071 (Decision Tree, Stacking Classifier) to 0.7143 (Logistic Regression).

In experiment 2, we followed the same process as in experiment 1; however, we used BERT Base Uncased instead of BioClinical BERT. As shown in table 6, the accuracy of our AD patient classifiers ranges from 0.6250 (AdaBoost) to 0.6786 (Logistic Regression).

In experiment 3, we performed an ablation study and assigned binary labels to the elements of each patient’s vector based on whether medSpacy was able to identify at least one sentence in each of the AD indicator categories that each vector element corresponds to. As shown in table 7, the accuracy across our AD patient classifiers ranges from 0.6964 (KNN) to 0.8036 (XGBoost).

## Discussion

### Sentence Classification Results

Our MLP sentence classifiers achieved accuracies between 0.7143 (classifier 3 w/ BioClinical BERT Embeddings) and 0.9814 (classifier 1 w/ BioClinical BERT Embeddings). If we applied the most accurate classifier from the aggregate of tables 3 and 4, we would achieve accuracies of 0.9814 for identifying sentences that directly suggest the patient has AD, 0.8776 for sentences that suggest hay fever, 0.7321 for sentences that suggest atopic allergies, 0.9234 for sentences that suggest eczema/rashes, 0.8955 for sentences that suggest dry/itchy skin, 0.8683 for sentences that suggest the use of non-asthma medications related to treating AD, 0.9288 for sentences that suggest the presence of asthma, and 0.8140 for sentences that suggest the use of asthma medications. Although classifier 3 only achieved accuracies of 0.7143 when using BioClinical BERT Embeddings and 0.7321 when using BERT Base Uncased embeddings, we believe that this is a reflection of the significantly lower number of training and testing samples we were able to identify in category 3 (as compared to other categories), as shown in table 2. Because we achieved accuracies between 0.8588 and 0.9814 in most cases and because our training and testing sets were both class-balanced, we believe these results are promising and indicate that our sentence classifiers could potentially be used to save time in a clinical setting during chart review for highlighting sentences that indicate patients have AD when recruiting for clinical trials.

One hypothesis we had made earlier was that using BioClinical BERT sentence embeddings to train sentence classifiers would provide better performance than using BERT Base Uncased sentence embeddings due to the clinical setting of our data. Given the results in tables 3 and 4, we determined that this was not the case in the context of sentence classification, as BioClinical BERT sentence embeddings yielded better performance in 4 of the 8 cases (categories 1, 2, 6, and 7 which represent mentions of AD, hay fever, asthma, and non-asthma medications), and BERT Base Uncased embeddings provided better performance in the other 4 of 8 cases (categories 3, 4, 5, and 8 which represent mentions of allergies, eczema or rashes, dry or itchy skin, and asthma medications). We believe this could be because embeddings for more commonly used concepts like rashes and dry/itchy skin could be better represented by general domain embeddings (BERT Base Uncased) whereas specialized terms representing clinical concepts of AD and AD related diseases/medications are better represented by specialized clinical embeddings (BioClinical BERT).

### AD Phenotyping Results

Although our hypothesis that using clinical embeddings (BioClinical BERT) would provide better performance than using non-clinical embeddings (BERT Base Uncased) did not hold in the context of sentence classification, we can see that our hypothesis does hold in the context of patient phenotyping. Specifically, in tables 5 and 6 we observed that AD patient classification when using BioClinical BERT generally results in better performance than using BERT Base Uncased, with the exception that BERT Base Uncased provides better performance in the cases of Decision Trees, XGBoost, and the stacking classifier.

As part of our experimental design, we included an ablation study in experiment 3 so we could compare the difference in performance during patient phenotyping when removing the use of transformer-based models to create each patient’s vector representations. Looking at the results, we observe that accuracies range from 0.6071 to 0.7154 when using BioClinical BERT embeddings in table 5, accuracies range from 0.6250 to 0.6786 when using BERT Based Uncased embeddings in table 6, and accuracies range from 0.6964 to 0.8036 when removing the use of transformer-based models in table 7. We found that models in experiment 3 (our ablation study) generally outperform their corresponding counterparts in experiments 1 and 2 (our BERT experiments) across all 3 metrics (accuracy, precision, and recall) with the exception that the recall of KNN in experiments 1 and 2 are higher than the recall of KNN in experiment 3. We hypothesize that models in experiments 1 and 2 showed lower performance because errors from our sentence classifiers in earlier stages of the pipeline could have propagated to later stages of the pipeline during patient phenotyping. Because we used the max operator to aggregate probabilities that any given sentence in the patient record applies to each category, more sentences in each patient record would lead to a greater chance that an erroneous prediction with a high probability would lead to a false positive error in the creation of each patient’s vector representation in experiments 1 and 2.

Although there is a wide range in performance for our AD patient phenotyping algorithms, we believe that we have reached our goal of developing a system capable of AD patient phenotyping for clinical trial recruitment because table 7 shows promising results for accuracy, precision, and recall. Furthermore, our system can be used as a first step during AD clinical trial recruitment to filter out most patients who may not qualify for AD trials and therefore save valuable clinician time. We believe our pipeline is important and valuable because unlike other diseases such as influenza, COVID-19, and cancer, there is no gold-standard test result that can be used to determine when a patient has atopic dermatitis. Instead, clinicians must spend large amounts of time undergoing chart review to individually determine whether each patient has atopic dermatitis.

### Limitations

One limitation of our study was the small size of our dataset. Although we had a total of 1,926 patients in our dataset, only 137 of them were validated as having AD. We chose to use a class-balanced approach of sampling from our dataset, and therefore had a total of 274 patients (half with AD and half without AD) from which we created our training and testing datasets. This could lead to overfitting and therefore result in reduced performance on the testing set. Future work could involve either obtaining more data from patients with AD, as well as exploring the use of an imbalanced dataset but using a class-weighted loss function to counteract the class-imbalance.

A second limitation of our study was the input limit size of the large language models that were used. Both BERT Base Uncased and BioClinical BERT had an input limit of 512 tokens. This meant that any input text that was longer than 512 tokens would be ignored when training BERT. Consequently, we couldn’t simply directly concatenate all documents from each patient’s EHR and feed the tokenized documents of each patient into BERT with an added classification head for training as well as direct prediction of whether the patient has AD. Instead, we had to design a pipeline around distilling information from all documents in each patient’s EHR into a patient vector representation and then using this patient vector representation to train various classical ML algorithms for phenotyping the patient. Future work could involve exploring the use of other LLM’s that are suited for long inputs such as Longformer or Doc2Vec for predicting when a patient should be labeled as having AD.

A third limitation of our study was the list of AD indicators we selected. We didn’t consider additional AD indicators and we also did not consider the use of different combinations (or subsets) of the AD indicators selected. This is particularly relevant in considering that 1) our pipeline is intended to be used for identifying patients with AD, and 2) that one of our AD indicators (category 1) directly targets whether there is any given sentence in the patient’s record that mentions AD which could be in the context of a family history of AD, a potential (but not confirmed) diagnosis of AD, as well as a confirmed diagnosis of AD, among other possibilities. If this AD indicator is removed, then one interesting research question could be whether our pipeline is still able to maintain performance similarly to what it is currently able to achieve. Future work could involve assessing the performance impact from removing (or adding) the use of various AD indicators. We could then determine if our pipeline is relying too much on or overfitting to one or more indicators. Furthermore, we could also re-design our patient vector and separate the feature for category 1 (any sentence that mentions AD) into 3 separate indicators, whether there is 1) a family history of AD, 2) an affirmed diagnosis that the patient has AD, and 3) uncertainty of whether the patient has AD. Doing so could potentially improve precision.

### Potential Applications

Given the aforementioned results, we believe our AD classifier could be operationalized to facilitate reliable and efficient EHR chart review. For example, optimized or ensemble-based sentence classifiers could visually indicate AD indicators inline text reducing information foraging efforts by clinician abstractors. Additionally, AD phenotyping classifiers could indicate the strength of a patient match to UKWP criteria, exact or partial, based on AD indicator sentence classifications. Furthermore, ranking patient cases by match strength could reduce the number of cases reviewed to generate both case and matched controls.

### Conclusions

In conclusion, we developed a promising pipeline for phenotyping patients with AD. Our most accurate AD phenotyping algorithm discerned AD from non-AD patients with an accuracy of 0.8036, precision of 0.8400, and a recall of 0.7500 when trained using XGBoost in experiment 3 with a binary patient vector representation. We hope that our work reduces clinician burden during clinical trial recruitment. Future work can involve extending our patient phenotyping pipeline to other datasets and other diseases.

## Figures and Tables

**Figure 1.**
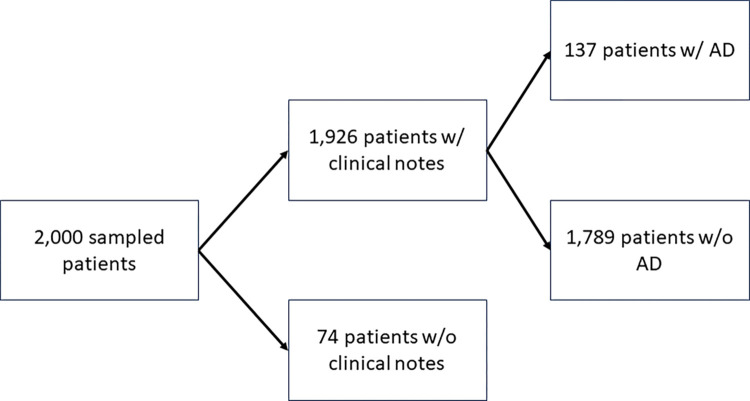
Results from dataset labeling

**Figure 3.**
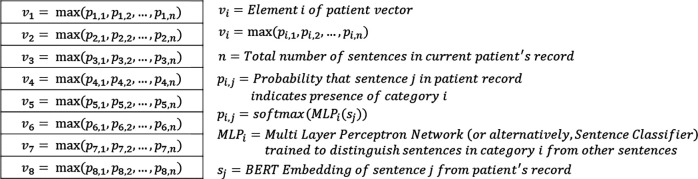
Patient vector representations of AD indicators in experiments 1 and 2

**Figure 4.**
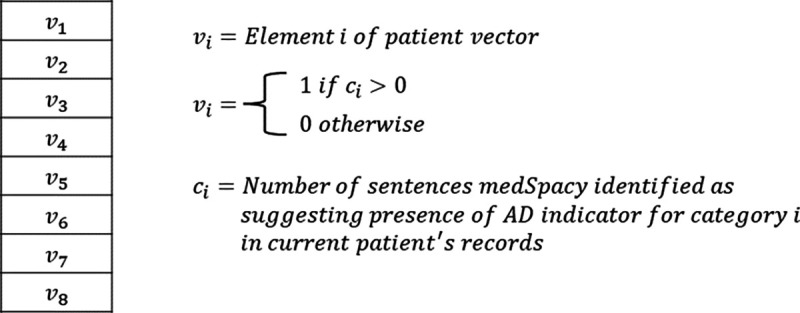
Patient vector representations of AD indicators in experiment 3

**Table 1.** Meaning of each patient vector element

Element	AD Indicator (Diagnostic Criteria)
1	EHR directly mentions patient has AD
2	Patient has hay fever allergies
3	Patient has atopic allergies
4	Patient has eczema or rashes
5	Patient has dry or itchy skin
6	Patient uses non-asthma medications related to treating AD
7	Patient has asthma
8	Patient uses asthma medications

**Table 2.** Training and testing dataset size for each classifier

Classifier	# of training samples	# of testing samples
1	2766	862
2	1302	392
3	532	168
4	9822	2454
5	1466	354
6	9114	2316
7	1596	520
8	4764	1070

**Table 3.** Accuracy of different multi-layer perceptron networks in discerning sentences by AD indicator categories using BioClinical BERT sentence embeddings

Classifier	AD Indicator	Accuracy
1	Direct mention of AD	0.9814
2	Mention of hay fever allergies	0.8776
3	Mention of atopic allergies	0.7143
4	Mention of eczema or rash	0.9193
5	Mention of dry or itchy skin	0.8898
6	Mention of non-asthma medications	0.8683
7	Mention of asthma	0.9288
8	Mention of asthma medications	0.7804

**Table 4.** Accuracy of different multi-layer perceptron networks in discerning sentences by AD indicator categories using BERT Base Uncased sentence embeddings

Classifier	AD Indicator	Accuracy
1	Direct mention of AD	0.9698
2	Mention of hay fever allergies	0.8673
3	Mention of atopic allergies	0.7321
4	Mention of eczema or rash	0.9234
5	Mention of dry or itchy skin	0.8955
6	Mention of non-asthma medications	0.8588
7	Mention of asthma	0.8327
8	Mention of asthma medications	0.8140

**Table 5:** AD Phenotyping Performance in Experiment 1 (BioClinical BERT)

Model	Accuracy	Precision	Recall
Logistic Regression	0.7143	0.7143	0.7143
SVM	0.6786	0.6667	0.7143
Decision Tree	0.6071	0.6154	0.5714
Random Forest	0.6786	0.6923	0.6429
KNN	0.6786	0.6923	0.6429
XGBoost	0.6250	0.6400	0.5714
AdaBoost	0.6429	0.6538	0.6071
Stacking Classifier	0.6071	0.6154	0.5714

**Table 6:** AD Phenotyping Performance in Experiment 2 (BERT Base Uncased)

Model	Accuracy	Precision	Recall
Logistic Regression	0.6786	0.6667	0.7143
SVM	0.6607	0.6452	0.7143
Decision Tree	0.6429	0.6429	0.6429
Random Forest	0.6429	0.6538	0.6071
KNN	0.6607	0.6667	0.6429
XGBoost	0.6429	0.6538	0.6071
AdaBoost	0.6250	0.6400	0.5714
Stacking Classifier	0.6607	0.6800	0.6071

**Table 7:** : AD Phenotyping Performance in Experiment 3 (Binary Vector Encoding)

Model	Accuracy	Precision	Recall
Logistic Regression	0.7679	0.7586	0.7857
SVM	0.7857	0.7857	0.7857
Decision Tree	0.7857	0.7667	0.8214
Random Forest	0.7679	0.8261	0.6786
KNN	0.6964	0.7391	0.6071
XGBoost	0.8036	0.8400	0.7500
AdaBoost	0.7857	0.7857	0.7857
Stacking Classifier	0.7500	0.7500	0.7500

## Abbreviations

AD: atopic dermatitis
BERT: Bidirectional Encoder Representations from Transformers
EHR: Electronic Health Records
ICD: International Classification of Disease
UKWP: United Kingdom Working Party
HR: Hanifin and Rajka
AI: Artificial Intelligence
NLP: Natural Language Processing
ML: Machine Learning
MLP: Multi-layer Perceptron
ReLU: Rectified Linear Unit
SGD: Stochastic Gradient Descent
KNN: K-Nearest Neighbors
XGBoost: Extreme Gradient Boosting
AdaBoost: Adaptive Boosting
SVM: Support Vector Machines
TP: True Positive
TN: True Negative
FP: False Positive
FN: False Negative
